# Protective Effects of Fucoidan on Aβ25–35 and d-Gal-Induced Neurotoxicity in PC12 Cells and d-Gal-Induced Cognitive Dysfunction in Mice

**DOI:** 10.3390/md15030077

**Published:** 2017-03-16

**Authors:** Hengyun Wei, Zixiang Gao, Luping Zheng, Cuili Zhang, Zundong Liu, Yazong Yang, Hongming Teng, Lin Hou, Yuling Yin, Xiangyang Zou

**Affiliations:** 1 Department of Biotechnology, Dalian Medical University, Dalian 116044, China; weihengyun@126.com (H.W.); 18641548759@163.com (Z.G.); biot_zhangcl@sina.com (C.Z.); harryblood1989@126.com (Z.L.); yangyazong123@126.com (Y.Y.); thm0529@126.com (H.T.); 2 College of Integrative Medicine, Dalian Medical University, Dalian 116044, China; zzllpp311@163.com; 3 College of Life Sciences, Liaoning Normal University, Dalian 116081, China; houlin01@126.com

**Keywords:** fucoidan, Alzheimer’s disease, neuroprotection, apoptosis, amyloid-β peptide

## Abstract

Alzheimer’s disease (AD) is a chronic neurodegenerative disease which contributes to memory loss and cognitive decline in the elderly. Fucoidan, extracted from brown algae, is a complex sulfated polysaccharide and potential bioactive compound. In this study, we investigated whether fucoidan protects PC12 cells from apoptosis induced by a combination of beta-amyloid 25–35 (Aβ25–35) and d-galactose (d-Gal), and improves learning and memory impairment in AD model mice. The results indicated that fucoidan could inhibit the release of cytochrome c from the mitochondria to cytosol and activation of caspases, and increase the expression of apoptosis inhibitor proteins (IAPs), including livin and X-linked IAP (XIAP) in PC12 cells damaged by Aβ25–35 and d-Gal-induction. Fucoidan reversed the decreased activity of acetylcholine (ACh) and choline acetyl transferase (ChAT), as well as the increased activity of acetylcholine esterase (AChE), in AD model mice induced by infusion of d-Gal. Furthermore, fucoidan improved antioxidant activity in vitro and in vivo by activation of superoxide dismutase (SOD) and glutathione (GSH). These results suggested that fucoidan could protect PC12 cells from apoptosis and ameliorate the learning and memory impairment in AD model mice, which appeared to be due to regulating the cholinergic system, reducing oxidative stress, and inhibiting the caspase-dependent apoptosis pathway.

## 1. Introduction

Alzheimer’s disease (AD), accounting for 60%–70% of cases of dementia all over the world, is associated with memory loss and cognitive decline in the elderly [1]. AD is the most common pathogenesis of age-related dementia, which is one of the leading causes of death for those aged 65 years and older [2]. It is a chronic and progressive neurodegenerative disorder characterized by degeneration and loss of neurons in the brain that usually begins with memory loss simultaneously [3]. Though the pathogenesis of AD is still unknown, it has been proved that accumulation of beta-amyloid (Aβ) fibrillar deposition in senile plaques and neurofibrillary tangles lesions in specific areas of brain correlates with the progression of cognitive dysfunction in AD patients [4,5,6]. The Aβ25–35 aggregates located around neurons not only have a direct toxic effect on the neurons, but also enhance the susceptibility of neuronal cells to free radicals, oxidative stress, and other harmful factors, leading to apoptosis of neurons [7,8]. Therefore, the inhibition of Aβ-induced neuronal apoptosis may provide a plausible approach for AD prevention and treatment.

Fucoidan is a sulfated fucose-containing polysaccharide extracted from brown algae and is a potential natural product for biological activities. It has been reported that fucoidan possesses a wide variety of biological activities, including antioxidant and anti-apoptosis activities [9]. Although several studies on the biological activities of fucoidan have been performed with particular focus on its antioxidant and anti-apoptosis activity, the neuroprotective effect of fucoidan is still unknown.

There are two models usually subjected to the investigation of pathogenesis and treatment of AD. d-Gal is a kind of monosaccharide. The high level of a d-Gal metabolite, galactitol, in brain cannot be metabolized and accumulates in neurons, which damages the balance of osmotic pressure and cell morphology, leading to loss of neurons and aging, even AD [10]. In addition, the release of free radicals in the metabolism of d-Gal also accelerates the aging progression. Aβ deposition in senile plaque formation results in cell death and vascular lesions, and eventually leads to aging and cognitive dysfunction. In this study, the combination of Aβ25–35 and d-Gal was used to establish an elaborate AD model for imitating the physiological state in vitro. PC12 cells, originally derived from a pheochromocytoma of rat adrenal medulla [11], were used to evaluate the antioxidant and anti-apoptosis effects of fucoidan on PC12 cells damaged with Aβ25–35 and d-Gal. We speculate that fucoidan may play a protective role in PC12 cells by reducing apoptosis and ameliorating the learning and memory impairment in AD model mice. We further evaluated the anti-apoptosis activity of fucoidan, which is derived from *Undaria pinnatifida* sporophylls, on PC12 cells in vitro. Moreover, we also established a mouse AD model to evaluate the protective effect of fucoidan on improving the learning and memory impairment induced by d-Gal in AD model mice, and revealed the potential protective mechanisms, which were significant regulation of the cholinergic system, reduction of oxidative stress, and inhibition of the caspase-dependent apoptosis pathway.

## 2. Results and Discussion

### 2.1. Fucoidan Improves the Viability of PC12 Cells Damaged by Aβ and d-Gal

**Figure 1:**
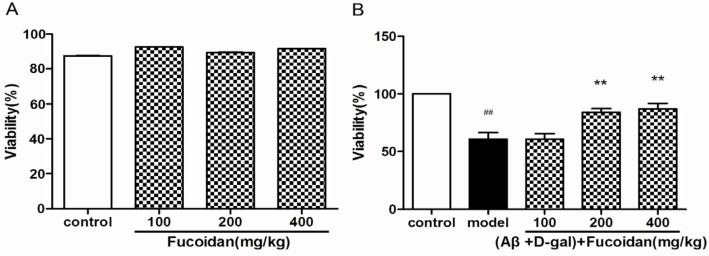
Effects of fucoidan on cytotoxicity and viability in PC12 cells injured by beta-amyloid (Aβ) and d-Gal. (**A**) PC12 cells were treated with different concentration of fucoidan (100, 200, and 400 µg/mL) for 24 h; no significant cytotoxicity was observed; (**B**) fucoidan (100, 200, and 400 μg/mL) can protect the cells injured by Aβ (25 μM) + d-Gal (10 mM). Data are presented as mean ± SD. * *p* < 0.05 and ** *p* < 0.01 compared with model, ## *p* < 0.01 compared with control (One-way ANOVA).

To verify the cytotoxic and neuroprotective effects of fucoidan for PC12 cells, cellviability assay was performed by the MTT method. The results indicated that fucoidan (100, 200, and 400 µg/mL, for 24 h) has no significant cytotoxicity (Figure 1A). The cell viability was markedly decreased in PC12 cells damaged by a combination of Aβ and d-Gal, while fucoidan effectively protected the cells compared with model (Figure 1B).

### 2.2. Fucoidan Prevents Apoptosis in PC12 Cell Induced by Aβ and d-Gal 

**Figure 2:**
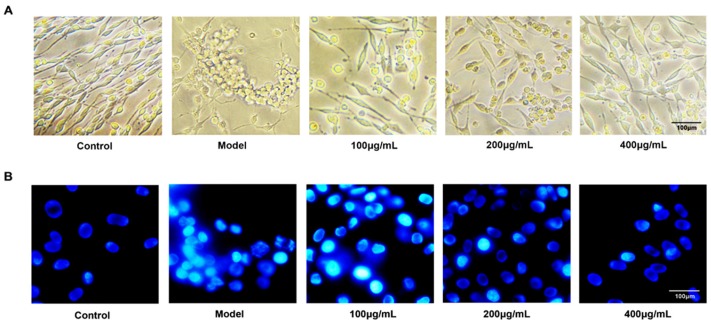
Morphology and fluorescent chemical staining of PC12 cells. (**A**) The changes of morphology of PC12 cells in different groups under light microscope observation. Fucoidan attenuated the morphological changes induced by Aβ + d-Gal, which resulted in decreased and shortened nerve filaments and cell aggregation. (**B**) Hoechst 33258 staining of PC12 cells injured by Aβ and d-Gal with different concentration of fucoidan (100, 200, 400 μg/mL). Magnification scale is 400×.

Fluorescent chemical staining and flow cytometry were performed to investigate the anti-apoptosis effect of fucoidan on PC12 cells injured with Aβ + d-Gal. The result showed that Aβ and d-Gal resulted in decreased and shortened nerve filaments and cell aggregation, and fucoidan reversed the morphological changes (Figure 2A). Hoechst 33258 staining further showed fucoidan can efficiently prevent chromatin condensation (Figure 2B). 

**Figure 3:**
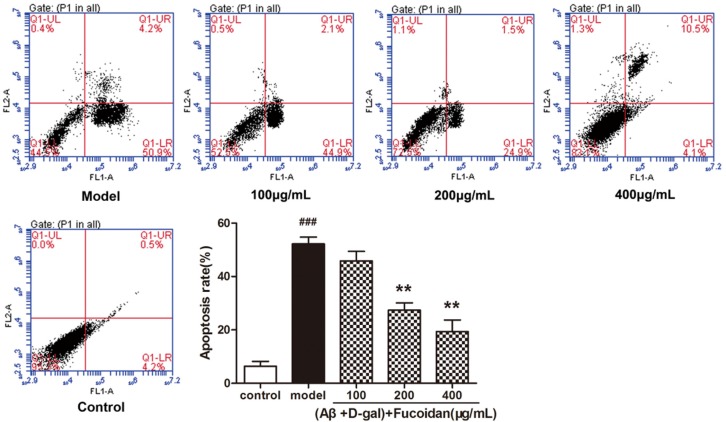
Flow cytometry examined apoptosis of PC12 cells. PC12 cells were treated with different concentrations of fucoidan (100, 200, 400 μg/mL) and Aβ and d-Gal (25 μM + 10 mM). Cell apoptosis was identified by annexin-V/propidium iodide (PI) double-staining assay. LL, LR, UR, and UL denote viable (live), early apoptotic, late apoptotic, and necrotic regions, respectively. Data are presented as mean ± SD. * *p*< 0.05 and ** *p*< 0.01 compared with model cells, ### *p*< 0.001 compared to control (one-way ANOVA).

Flow cytometry was used to detect the effect of fucoidan on apoptosis in PC12 cells. The result showed that the fucoidan reduced the apoptosis rate of PC12 cells damaged by Aβ and d-Gal. With the increase of fucoidan concentration (100, 200, 400 μg/mL), the proportion of apoptotic cells (right-lower quadrant) significantly reduced from 50.9% in the model to 44.9%, 24.9%, and 4.1%, respectively (Figure 3).

### 2.3. Effect of Fucoidan on Levels of Apoptosis-Related Proteins in PC12 Cells 

**Figure 4:**
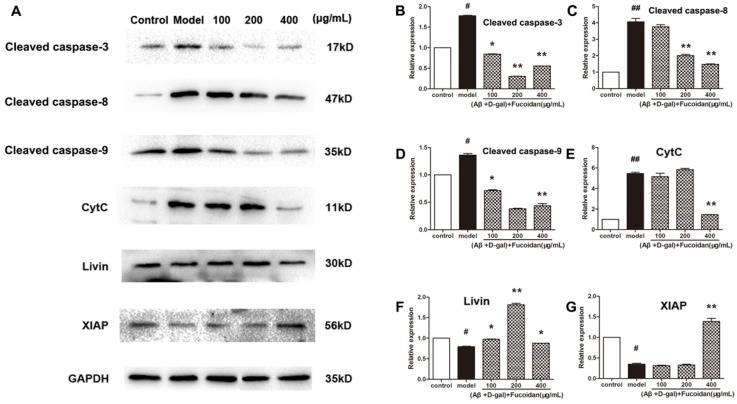
Effect of fucoidan on caspases activation and expression of apoptosis inhibitor proteins (IAPs) in PC 12 cells damaged by Aβ and d-Gal. (**A**) The levels of apoptosis-related proteins, including cleaved caspase-3, caspase-8, and caspase-9, livin and X-linked IAP (XIAP) protein levels, and cytochrome c (cyt c) content in the cytoplasm. Western blotting analysis of apoptosis-related proteins levels; (**B**–**D**) cleaved caspase-3, caspase-8, and caspase-9 levels; (**E**) cyt c content in the cytoplasm; (**F**–**G**) expression of livin and XIAP proteins in PC12 cells injured by Aβ and d-Gal. Protein quantification is expressed as the mean ± SD of three time independent experiments. * *p* < 0.05 and ** *p* < 0.01 compared with model, # *p* < 0.05 and ## *p* < 0.01 compared with control (one-way ANOVA).

To verify whether fucoidan intervened apoptosis via the caspase-dependent pathway, we measured the level of cleaved caspase-3, cleaved caspase-8, and cleaved caspase-9, and release of cytochrome c (cyt c). As shown in Figure 4, compared to model, fucoidan decreased the levels of cleaved caspase-3, caspase-8, and caspase-9 (Figure 4B–D), and prevented the release of cyt c from the mitochondria to cytosol (Figure 4E). Two members of inhibitors of apoptosis protein family, livin and X-linked apoptosis inhibitor protein (XIAP), suppress apoptosis by inhibiting the activation of caspases. These proteins were upregulated by fucoidan in damaged PC12 cells (Figure 4F–G). 

### 2.4. Effect of Fucoidan on SOD Activity and GSH Content in PC12 Cells

**Figure 5:**
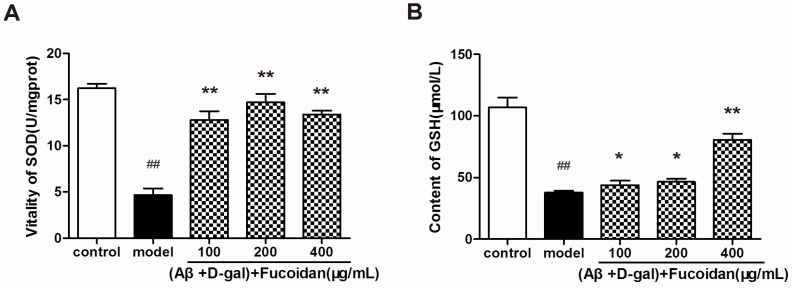
Effect of fucoidan on superoxide dismutase (SOD) activity and glutathione (GSH) content in PC12 cells damaged by Aβ and d-Gal. (**A**) SOD enzyme activity in different treatments of PC12 cells. (**B**) GSH content was increased in PC12 cells treated with fucoidan compared with that of model. Data are presented as mean ± SD. * *p* < 0.05 and ** *p* < 0.01 compared with model, ## *p* < 0.01 compared with control (one-way ANOVA).

The superoxide dismutase (SOD) enzyme activity and glutathione (GSH) content were detected in PC12 cells by commercial colorimetric assays. The results showed that, with fucoidan treatment, SOD activity (Figure 5A) and GSH content (Figure 5B) were significantly increased in PC12 cells damaged by Aβ and d-Gal compared with that of model cells.

### 2.5. Effects of Fucoidan on Learning and Memory Improvements of AD Model Mice

d-Gal-injected mice (model group) showed spatial learning and memory impairment by using the Morris water maze test, as indicated in Table 1; the escape latency of model mice was longer than that of control mice. However, AD model mice with the administration of fucoidan (100 and 200 mg/kg on day 2–6, 50 mg/kg on day 4–6) exhibited shortened escape latency of the training trials (*p* < 0.05), which suggested that fucoidan could ameliorate and improve the spatial learning and memory in mice impaired by d-Gal injection. 

### 2.6. Effect of Fucoidan on Cholinergic System and Biochemistry Indexes In Vivo

**Figure 6:**
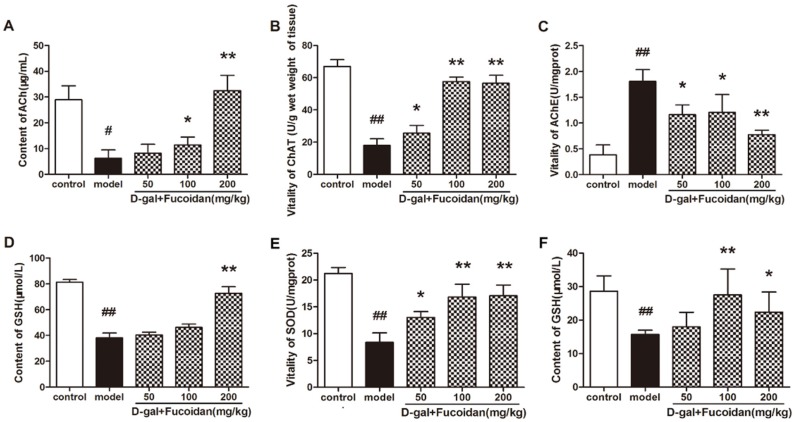
Effect of fucoidan on the levels of components of the cholinergic system and biochemistry indexes in brain tissue and blood serum in vivo. (**A**–**C**) Acetylcholine (ACh) content and choline acetyl transferase (ChAT) and acetylcholine esterase (AChE) activities; (**D**) GSH content in brain tissue of each group of mice; (**E**) SOD enzyme activity and (**F**) GSH content in blood serum. Data are presented as mean ± SD from 8 mice in each group. * *p* < 0.05 and ** *p* < 0.01 compared to model, # *p* < 0.05 and ## *p* < 0.01 compared to control (one-way ANOVA).

Considering that the cholinergic system plays a crucial role in cognitive function, we tested the effect of fucoidan administration on acetylcholine (ACh) content, and choline acetyl transferase (ChAT) and acetylcholine esterase (AChE) enzyme activities in mice brain homogenate. The levels of ACh content and ChAT activity were increased significantly, while the AChE activity was decreased in fucoidan-treated mice compared with that of model mice (*n* = 8) (Figure 6A–C). Meanwhile, the brains of fucoidan-treated mice exhibited a higher content of GSH than that of the model mice (Figure 6D). The SOD activity and GSH content in the blood serum of mice treated with fucoidan were obviously increased compared with that of model (Figure 6E,F). 

### 2.7. Fucoidan Protects Hippocampal Neurons Impaired by d-Gal In Vivo

**Figure 7:**
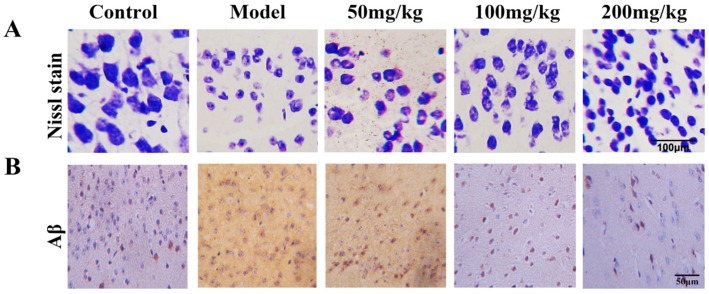
Nissl staining of hippocampus cornu ammonis (CA1) neurons in mice damaged by d-Gal injection. (**A**) The fucoidan (50, 100, 200 mg/kg) ameliorated morphological disorder of hippocampus CA1 neurons, and maintained the density of neurons compared to model mice. (**B**) Aβ staining of CA1 neurons in d-Gal-induced AD model mice.

To determine whether fucoidan can protect d-Gal-induced cell impairment, we performed Nissl staining to analyze the density and shape of neurons in the hippocampus cornu ammonis (CA1) of mice. In control mice, hippocampal neurons were normal without degeneration or pyknosis, and showed abundant Nissl bodies in cytoplasm. In d-Gal-injected mice, patterns of neurons were disordered, showing pyknosis, reduced volume, and a hyperchromatic intercellular space. However, fucoidan ameliorated morphological disorder of hippocampal neurons, and the density of neurons was also increased significantly in AD model mice (Figure 7A). In order to further confirm whether fucoidan can decrease Aβ deposition in the hippocampus CA1 in AD mice, we carried out immunohistochemistry staining. The results indicated that Aβ deposition, which is the character similar to that of Alzheimer’s disease, was much more in model mice, and fucoidan treatment resulted in a decrease in CA1 (Figure 7B).

### 2.8. Discussion

Aβ, namely senile plaques and cerebral amyloid angiopathy, is the major histopathological feature in AD. Aβ deposition causes the degeneration and apoptosis of nerve cells and leads to cognitive impairment and memory loss. Aβ has been used as a toxic agent to neurons both in vitro and in vivo [12,13]. As galactose metabolites, galactitol cannot be absorbed and is further deposited in neurons, which leads to cell osmotic pressure changing, cell swelling, membrane lipid impairment, cranial nerve degeneration, and, eventually, results in aging. In this study, we used the d-galactose damage model in vivo. On the basis of other researchers’ studies, we improved on the model and designed the combination of Aβ and d-Gal as a cell injury model in vitro. Our results showed that fucoidan has no cytotoxicity to PC12 cells, while it improves the viability and prevents apoptosis in PC12 cells injured by Aβ and d-Gal. 

Neurofilaments’ low polymerization and neuronal tangles caused by excessive neurofilament protein phosphorylation is a remarkable feature of AD [14,15]. These phenomena render neurofilaments liable to lose the ability to interact with microtubules as well as the stabilizing effect on the microtubules, and gather to form spiral wires [16,17]. These changes cause an intracellular anomaly, cytoskeleton damage, morphology changes, and apoptosis [18]. There were fewer and shorter neurofilaments around the injured cells, and the cells clustered into a mass, as observed under light microscopy. However, the fucoidan reversed the deterioration by maintaining the fusiform appearance and levels of neurofilaments of cells. Hoechst 33258 staining was performed to check the apoptosis of PC12 cells in presence or absence of fucoidan by flow cytometry (FCM). The chromosomes of the model group’s cells exhibited shrinkage characteristics in apoptotic cells by showing aggregated high fluorescence intensity. The shrinkage of chromosomes was attenuated in the fucoidan treatment group by showing diffuse, homogeneous, low fluorescence intensity. These results implied that fucoidan could not only maintain the morphology of PC12 cells but also increase the viability of cells and protect cells from apoptosis. We speculated the fucoidan might interact with several signaling pathways to resist apoptosis induced by Aβ and d-Gal.

Apoptosis, also known as programmed cell death, results from stimulus of internal and external environment changes or cell death signals that can activate the death receptor, causing nuclear DNA damage [19]. The caspase-dependent apoptotic pathway can be initiated through two pathways: extrinsic pathway by initiator caspase-8 and intrinsic pathway by initiator caspase-9 [20]. Whether it is by way of intrinsic or extrinsic pathway, apoptosis is induced by activating executioner caspase-3, which then kills the cell by degrading proteins indiscriminately. In this paper, the regulation of the caspase-dependent apoptotic pathway by fucoidan was investigated. We found fucoidan could upregulate the expressions of two apoptosis inhibitor proteins, XIAP and livin, which could mediate the activation of apoptosis pathways, both intrinsic and extrinsic [21]. It is well known that caspase-3 has proved to play an important role in the process of aging induced by Aβ. In postmortem AD brains, there is an increase in mRNA expression of multiple caspases, including caspases-3, 8, and 9, compared with control brains [22,23]. At the protein level, high levels of caspase-3 and caspase-9 could be found in synaptosomal fractions from AD brains [24,25]. Our results showed that the levels of cleaved caspase-8, caspase-9, and caspase-3 were downregulated by the intervention of fucoidan in PC12 cells. Therefore, we hold the opinion that fucoidan could inhibit apoptosis induced by Aβ and d-Gal by upregulating the expression of XIAP and livin proteins, preventing cyt c release from mitochondrion to cytosol and activation of caspases. 

Oxidative stress refers to imbalance of cell oxidation and antioxidation ability, leading to cell function disorder. If oxygen free radicals in the body cannot be cleared in time, accumulation of oxygen free radicals causes oxidative damage, mitochondrial dysfunction, and the occurrence of apoptosis. Both Aβ and d-Gal could produce free radicals, reduce the activity of antioxidant enzymes, and lead to mitochondrial dysfunction and cell death by interacting with the mitochondrial apoptosis pathway [26,27]. Previous studies have shown that fucoidan has an antioxidant effect, and a strong ability to remove superoxide radical and hydroxyl free radical effect [28]. The present study proved that fucoidan could improve the antioxidant capacity of PC12 cells by increasing the activity of antioxidant enzyme SOD and bioactive peptide GSH. In addition, the cytoplasm cyt c decreased under the pretreatment of fucoidan, which indicated the integrity of mitochondria was maintained. These results suggested that fucoidan can exhibit neuroprotective effects by activating endogenous antioxidants and resisting oxidative stress induced by Aβ and d-Gal, and then inhibiting apoptosis.

The water maze test was performed and the results showed that fucoidan improved the learning and memory capability in AD model mice. AChE is crucial for the regulation of the nervous system, and can terminate the release of ACh into the synaptic cleft. ChAT is involved in the synthesis of ACh. Moreover, the cognitive dysfunction in the AD patients is obviously related to decrease of ChAT enzyme activity and loss of cholinergic neurons, so AChE and ChAT play important roles in regulating ACh content [29]. In this study, the injection of d-Gal into mice resulted in significantly increased activity of AChE and decreased activity of ChAT, as well as a significant decrease in ACh content in brain tissue of mice, which was conducive to cognitive dysfunction. In contrast, the experimental results showed that fucoidan markedly reversed the enzyme activities of ChAT and AChE. ChAT activity was raised and AchE activity was decreased, which was beneficial to the synthesis of ACh. Furthermore, the hippocampus was studied as part of a brain system responsible for spatial learning and memory. Our results showed that fucoidan rescued the spatial learning and memory ability in mice injured by d-Gal. The Nissl staining implied that fucoidan might improve learning and memory ability by maintaining the density and shape hippocampus CA1 neurons in mice, and decrease Aβ deposition in these cells. These are thought to be important factors for improving the learning and memory ability by fucoidan in AD mice. How or whether fucoidan enters the central nervous system (CNS) has been unclear. The mechanism of the neuroprotection may partly depend on its antioxidative potential. 

## 3. Materials and Methods

### 3.1. Reagents and Antibodies

DMEM high-glucose medium, penicillin–streptomycin, and fetal bovine serum (FBS) were purchased from HyClone Cell Culture (Thermo Fisher Scientific, Carlsbad, CA, USA). Aβ25–35 and d-Gal were purchased from Sigma (St. Louis, MO, USA). Rabbit anti-caspase-3, caspase-8, and caspase-9 antibodies were purchased from Proteintech (Chicago, IL, USA). Rabbit anti-livin and XIAP were purchased from Boster (Wuhan, China). Mouse anti-GAPDH antibody was purchased from Kang Chen Bio-tech (Shanghai, China). Horseradish peroxidase-conjugated anti-rabbit and anti-mouse IgG were purchased from Thermo Fisher Scientific (Carlsbad, CA, USA).

### 3.2. Purification and Analyses of Fucoidan

The crude fucoidan used in this study was kindly provided from Dalian Aquaculture Group Co., Ltd. (Dalian, China). Fucoidan was collected from the coast of Dalian, Liaoning province, China, in March 2012. Crude fucoidan was extracted with distilled water at 80 °C for 1 h [30]. The fucoidan was purified as previously reported [31]. The fucoidan (purity > 90%) contained 41.48% carbohydrate, 12.69% sulfates, and 13.90% uronic acid; the protein content was merely 0.13%. Its molecular weight was approximately 26.61 × 10^4^ Da.

### 3.3. Aβ25–35 and d-Gal Preparation

Aβ25–35, which is a chemosynthesis product of toxic peptide fragment designed based on natural amyloid protein, was dissolved in deionized water and incubated with constant oscillation at 37 °C for 3 days to induce its aggregation [32]. After aggregation, the solution of Aβ25–35 was stored at −20 °C. The stock solution was diluted to working concentration immediately and added to cell culture medium before use. d-Gal (10 mM) solution was prepared by dissolving 18.016 mg d-Gal in 10 mL deionized distilled water. We next filtered the solution by 0.22 μm ultrafiltration membrane and then stored it at 4 °C.

### 3.4. Animals

Male ICR mice (20 ± 2 g) were obtained from the Experimental Animal Center of Dalian Medical University (SPF grade). The animals were housed in cages under hygienic conditions and placed in a controlled environment with a 12 h light/dark cycle at 22 ± 2 °C and 40%–70% humidity for 7 days before the experiment. The animals were allowed a commercial standard mice cube diet and water *ad libitum*. All animal experiments were carried out in accordance with the National Institutes of Health Guide for the Care and Use of Laboratory Animals and were approved by the animal care and use committee of Dalian Medical University.

### 3.5. Cell Culture and Treatment

The PC12 cells were obtained from the KeyGEN Biotech Co., Ltd. (Nanjing, China). The cells were routinely maintained in medium containing 10% (*v/v*) heat-inactivated fetal bovine serum (FBS) and 5% horse serum, penicillin (100 U/mL), and streptomycin (100 mg/mL) at 37 °C in a humidified incubator containing 5% CO_2_. Culture medium was changed every other day. After 24 h, cells were pretreated in the absence or presence of fucoidan (100, 200, and 400 μg/mL) and cultured for 24 h, followed by incubating with Aβ25–35 (25 μM) + d-Gal (10 mM) for an additional 48 h.

### 3.6. Cell Viability Assay

Cell viability was estimated by the MTT reduction assay. In brief, PC12 cells (1.0 × 10^5^ cells/well, in 200 µL medium) seeded in 96-well plates were exposed to fucoidan at given concentrations (100, 200, 400 μg/mL) for 24 h after treatment of Aβ and d-Gal (25 μM + 10 mM). MTT (20 μL) was added to each well and incubated for 4 h, and the absorbance at 490 nm was measured using a microplate reader (Thermo Fisher). Cell viability was expressed as the percentage of the absorbance of treated cells relative to the absorbance of control cells.

### 3.7. Light Microscope Observation

PC12 cells were treated by different concentration of fucoidan (100, 200, 400 μg/mL) and Aβ + d-Gal (25 μM + 10 mM) for 48 h, then changes inmorphology of PC12 cells were observed under light microscope (Olympus, Tokyo, Japan).

### 3.8. Hoechst 33258 Staining

The cells were treated with different concentrations of fucoidan (100, 200, 400 μg/mL) and Aβ + d-Gal (25 μM + 10 mM) for 48 h, incubated with Hoechst 33258 (4 μg/mL) for 30 min, fixed for 10 min in 4% paraformaldehyde (PFA), and then observed by DMI-4000B inverted fluorescence microscopy (Leica, Solms, Germany).

### 3.9. Apoptosis Assay

PC12 cells were seeded into cell culture flasks, allowed to adhere for 24 h, and treated with fucoidan (100, 200, and 400 μg/mL) and Aβ + d-Gal (25 μM + 10 mM) for 48 h, respectively. The cells were collected and washed twice with chilled phosphate-buffered saline (PBS) and subjected to staining with annexin V–FITC labeling solution (annexin V–fluorescein in binding buffer containing propidium iodide (PI) (KeyGen, Nanjing, China)) according to the manufacturer’s protocol. Apoptotic cells were analyzed by using a FACS Calibur flow cytometry.

### 3.10. Western Blot Analysis

Western blot was performed to evaluate the effects of fucoidan on the expression of apoptosis factors. Cells treated with different concentrations of fucoidan and Aβ + d-Gal were collected and then washed twice with chilled PBS and lysed in RIPA buffer (Sigma, St. Louis, MO, USA). Equal amounts of protein extract were subjected to 12% SDS-PAGE gels and transferred to nitrocellulose (NC) membranes (Solarbio, Beijing, China). The primary antibodies, including dilution conditions, are as follows: rabbit anti-caspase-3 (1:500), caspase-8 (1:300), caspase-9 (1:1000), cytochrome c (1:200), Livin (1:200), XIAP (1:200), and anti-GAPDH (1:5000) as an internal control for other proteins. Membranes were subsequently incubated with horseradish peroxidase (HRP)-conjugated secondary antibodies (1:15,000) (Thermo Fisher Scientific) for 1 h at room temperature. HRP-labeled antibodies bound to the membranes were detected by chemiluminescence.

### 3.11. Biochemistry Index of PC12 Cells

Cells treated with different concentration of fucoidan and Aβ + d-Gal were collected, washed twice with chilled PBS, and lysed in RIPA buffer as mentioned above. The supernate was used for the assays of SOD activity and GSH content, according to the manufacturer’s instructions (Jiancheng Bioengineering Institute, Nanjin, China).

### 3.12. Treatments in Animals

Forty male ICR mice were divided into the following five groups (8 animals per group): (I) Control: normal saline (NS); (II) Model: d-Gal (125 mg/kg, 0.2 mL); (III) d-Gal + fucoidan (50 mg/kg); (IV) d-Gal + fucoidan (100 mg/kg); (V) d-Gal + fucoidan (200 mg/kg). Mice of groups II–V were injected subcutaneously with d-Gal (125 mg/kg, 0.2 mL), and the control group mice were injected equal volume of NS, respectively. After 2weeks, the mice of groups III–V received fucoidan orally once daily for 21 days, and groups I and II received the same volume of NS. 

### 3.13. Morris Water Maze

A total of 40 mice were subjected to the Morris water maze test, which was performed as described previously [33] with some modifications. Training phase was performed two times daily for six days and escape latency (time of finding submerged platform) of each mice was observed. On each trial, the mice were allowed to swim until they found the platform and stay on the platform for an additional 10 s. However, the mice failing to find the platform within 120 s were placed on the platform manually for 10 s and the escape latency was recorded as 120 s.

### 3.14. Cholinergic System and Biochemistry Estimations 

After treatment with fucoidan, mice were weighed and blood was drawn from inner canthus. The global cerebrals were isolated and dried, and then weighed and prepared as a 10% tissue homogenate in ice-cold 0.9% saline solution. The homogenate was centrifuged (3500 rpm, for 15 min) and the supernatant was collected for detecting the content of ACh and GSH, and enzyme activities of AChE and ChAT. The blood was centrifuged (2000 rpm, for 10 min) and the serum was used for the assays of GSH content and SOD activity. The content of ACh and GSH, and activities of AChE, ChAT, and SOD detection assay kits were obtained from Jiancheng Bioengineering Institute (Nanjin, China). All operations were carried out following manufacturer’s instructions (www.njjcbio.com).

### 3.15. Nissl and Immunohistochemistry Staining

After the Morris water maze experiment, the mice were deeply anesthetized with 10% chloral hydrate. The brain was dissected and stored in 4% paraformaldehyde, before being dehydrated by the conventional alcohol gradient, embedded in paraffin, and cut into 4 μm thick coronal slices. Consecutive slices were mounted on clean slides treated by poly-l-lysine, incubated in a 60 °C oven for 2 h, and saved for later use. For Nissl staining, the sections were placed in xylene to dewax and alcohol for benzene removal. After washing in distilled water, the sections were stained in Nissl staining solution and incubated for 10 min at 37 °C. The sections were then soaked in 95% alcohol for 5 s and viewed carefully under a microscope while they were differentiated in 95% alcohol. Finally, they were dehydrated in 95% alcohol immediately after Nissl bodies appeared and then sealed with neutral balsam.

Immunohistochemistry staining was carried out using a streptavidin/peroxidase staining kit and anti-amyloid antibody (Boster Biotech, Wuhan, China) according to the manufacturer’s instructions.

### 3.16. Statistical Analysis

Data are expressed as mean ± standard deviation (SD). Significant differences were evaluated by one-way or two-way analysis of variance using Prism 5 (Version 5.04, GraphPad Software, Inc., La Jolla, CA. USA). Statistical significance was defined as *p* < 0.05 and *p* < 0.01. All experiments were performed at least three times for quantitative comparison.

## 4. Conclusion

Fucoidan showed potential neuroprotective effects against Aβ- and d-Gal-induced apoptosis in PC12 cells and d-Gal-induced learning and memory impairment in AD model mice. The mechanisms involved include regulating the cholinergic system, reducing oxidative stress, and inhibiting caspase and mitochondria apoptosis pathways. These results suggest that fucoidan might be a candidate neuroprotective agent against AD in aging conditions.

## Figures and Tables

**Table 1:** Effects of fucoidan on the Morris water maze task in Alzheimer’s disease (AD) model mice.

Groups	Dose (mg/kg)	Escape Latency Time (s)					
		1 Day	2 Day	3 Day	4 Day	5 Day	6 Day
Control		27.10 ± 8.66	23.07 ± 10.87	12.53 ± 7.03	11.93 ± 4.37	7.08 ± 1.53	7.36 ± 5.25
Model	0	73.51 ± 19.99 #	63.82 ± 23.19	54.09 ± 33.65	49.95 ± 29.40	47.19 ± 18.17 #	38.62 ± 19.51 #
Fucoidan	50	46.54 ± 9.56	41.54 ± 10.65	41.06 ± 15.55	36.24 ± 18.35 *	39.74 ± 17.68 *	28.52 ± 16.51 *
	100	39.46 ± 15.27 *	23.89 ± 13.83 **	15.95 ± 11.02 **	14.78 ± 11.98 **	14.24 ± 8.37 **	11.1 ± 7.09 **
	200	41.60 ± 16.70 *	21.61 ± 10.47 **	21.16 ± 12.28 **	18.12 ± 9.09 **	11.54 ± 4.58 **	9.87 ± 3.76 **

Note: data are presented as mean ± SD. # *p* < 0.05 compared to control; * *p* < 0.05, ** *p* < 0.01, compared to model. *n* = 8.

## Abbreviations

AD	Alzheimer’s disease;
Aβ25–35	beta-amyloid 25–35;
d-Gal	d-galactose;
ACh	acetylcholine;
AChE	acetylcholine esterase;
ChAT	choline acetyl transferase;
Cyt C	cytochrome C;
GSH	glutathione;
FCM	flow cytometry;
SOD	superoxide dismutase;
XIAP	X-linked IAP;
IAP	inhibitor of apoptosis protein.
